# A microfluidic multiplex proteomic immunoassay device for translational research

**DOI:** 10.1186/s12014-015-9101-x

**Published:** 2015-12-11

**Authors:** Jing Cao, Jesse Seegmiller, Naomi Q. Hanson, Christopher Zaun, Danni Li

**Affiliations:** Department of Laboratory Medicine and Pathology, University of Minnesota, Twin Cities, 420 Delaware Street SE, MMC 609, Minneapolis, MN 55455 USA

**Keywords:** Cytokine, Multiplex, Immunoassay, Microfluidics

## Abstract

**Objective:**

Microfluidic technology has the potential to miniaturize and automate complex laboratory procedures. The objective of this study was to assess a microfluidic immunoassay device, Simple Plex, which simultaneously measured IL-1β, TNF-α, IL-6, and IL-10 in serum samples. This assessment is important to understanding the potentials of this microfluidic device as a valuable tool in translational research efforts.

**Methods:**

We studied the operational characteristics of Simple Plex, and compared to other immunoassay systems including bead-based (i.e., Bio-Plex^®^ from Bio-Rad) and planar micro-spot based (i.e., Multi-Array from Meso Scale Discovery) multiplex assays. We determined imprecisions for each of the Simple Plex assays and evaluated the ability of Simple Plex to detect IL-1β, TNF-α, IL-6, and IL-10 in serum samples.

**Results:**

Simple Plex assays required 25 µL serum, and 1.5 h to run 16 samples per cartridge per instrument. Assay imprecisions, evaluated by measurement of 6 replicates in duplicate from a serum pool using three different cartridges, were less than 10 % for all 4 cytokine protein biomarkers, comparable to the imprecisions of traditional ELISAs. The Simple Plex assays were able to detect 32, 95, 97, and 100 % [i.e., percentages of the results within the respective analytical measurement ranges (AMRs)] of IL-1β, TNF-α, IL-6, and IL-10, respectively, in 66 serum samples.

**Conclusions:**

Simple Plex is a microfluidic multiplex immunoassay device that offers miniaturized, and automated analysis of protein biomarkers. Microfluidic devices such as Simple Plex represent a promising platform to be used in translational research to measure protein biomarkers in real clinical samples.

## Background

The technology of microfluidics is one that manipulates small volumes of fluid and flow that has the potential to miniaturize complex laboratory procedures [1, 2]. Microfluidic technology has been widely used in point-of-care (POC) devices for clinical diagnostics (e.g., iSTAT) [3–7]. Since these devices generally require small sample volumes there is much interest in applying microfluidic technology to areas outside of the traditional realms of POC diagnostics and into areas in translational research efforts such as the quantitative measurement of multiple protein biomarkers (multiplexing) [8–11]. One of the most widely used approaches for quantitative multiplexing of proteins is multiplex immunoassay [8]. There are several different platforms that are used to perform this type of analysis and some examples of this are: bead–based flow cytometry (e.g., Bio-Plex^®^ and Luminex^®^), and planar assays containing a defined array of capture microspots deposited in the bottom of the well (e.g., Multi-Array and Aushon Biosystem) [12]. While the above platforms have shown great utility in multiplexing quantitation, the microfluidics platform offers several advantages by its design. First, microfluidics allows for separate incubation chambers for every analyte; that is, each incubation chamber is limited to one antibody pair to react with its respective analyte. This prevents the potential issue of cross-reactivity (i.e., antigen cross-reacting to other antibody pairs) [13]; second, the consumption of reagent and sample volume is low due to miniaturized design, and therefore researchers are able to conserve precious reagents and samples [14]; third, microfluidics provides a means for automation where it may be able to automate sample preparation, incubation, and detection all on one device (or lab-on-a-chip) so that complex lab procedures can be minimized [1].

Despite all the potential advantages, multiplex immunoassays based on microfluidic technology have yet to be generally employed in translational research for measurement of protein biomarkers. This is likely due to that microfluidics by itself does not offer the sensitivity and specificity needed for measurement of proteins [15]. There has been interest in coupling microfluidics with nanotechnology, which would offer novel capture of antigens (selectivity) and improved detection (sensitivity) [16–20]. Recently, ProteinSimple has commercialized a microfluidic multiplexed immunoassay platform coupled with glass nanoreactors (GNRs) [21]. The objective of this study was to assess Simple Plex for measurement of the following protein biomarkers in patient serum samples: IL-1β, TNF-α, IL-6, and IL-10. We chose these cytokines for several reasons: (a) they are often measured simultaneously and involved in numerous acute and chronic disease conditions as part of balanced act of the immune system [22]; and (b) their concentrations can span wide dynamic ranges, from very low under non-pathological conditions to extremely high under acute inflammatory conditions, and therefore they can be very analytically challenging to measure simultaneously [12]. This assessment was key to understanding the potentials of this microfluidic multiplexed immunoassay platform and was a valuable tool to determine efficacy for translational research efforts in the quantitative measurement of multiple protein biomarkers in real clinical samples.

## Methods

### Clinical samples

Serum samples were randomly selected from the Endocrinology Laboratory at the University of Minnesota Medical Center, Fairview. Samples were stored frozen at −80 °C. The study protocol was reviewed and approved by the University of Minnesota Institutional Review Board (IRB).

### Multiplex protein biomarker measurement by Simple Plex

Protein biomarkers, IL-1β, TNF-α, IL-6, and IL-10, were measured in the serum samples using Simple Plex (ProteinSimple, San Jose, CA) following the manufacturer’ instruction. The work flow of the Simple Plex assays is that: (a) a test cartridge is primed with samples with each sample split into 4 channels (i.e., IL-1β, TNF-α, IL-6, and IL-10) to react with their respective antibodies immobilized on glass nanoreactors; (b) after sample incubation, circuits in the cartridge are cleaned with wash buffer, and biotinylated detection antibody solutions are individually pumped into their respective channels to bind to protein analyte captured on the GNRs; (c) after incubation, unbound detection antibodies are washed away and a detection solution (i.e., streptavidin DyLight 650) is flowed into all 4 channels to conjugate with the biotinylated detection antibodies; (d) the detection solution is washed away; detection fluorophores (i.e., DyLight 650) are excited with a 631 nm laser; and the fluorescence signals are read with a charge-coupled device (CCD) camera. The signals are used for quantification based on master calibrator curves provided by the manufacturer. Simple Plex uses 25 µL of serum for measurement of the four cytokines. Precisions of the Simple Plex assays were evaluated by duplicate measurement of a pooled serum sample on three different Simple Plex cartridges. Sixty-six serum samples were measured using Simple Plex cartridges from the same lot on 2 consecutive days.

### Comparison of Simple Plex to ELISAs and other multiplex immunoassays

We measured IL-1β, TNF-α, IL-6, and IL-10 in these 66 samples using ELISAs (Bio-Techne, formerly R&D systems, Minneapolis, MN) and studied the operation characteristics of ELISAs from our own experiments. Although we did not measure IL-1β, TNF-α, IL-6, and IL-10 in these 66 samples using the Bio-Plex^®^ and Multi-Array assays, we included the operational characteristics of the BioPlex^®^ and Multi-Array assays, which were derived from our previous experiences in using these systems [23–25], in the comparison to those of the Simple Plex and ELISAs.

## Results

**Fig. 1:**
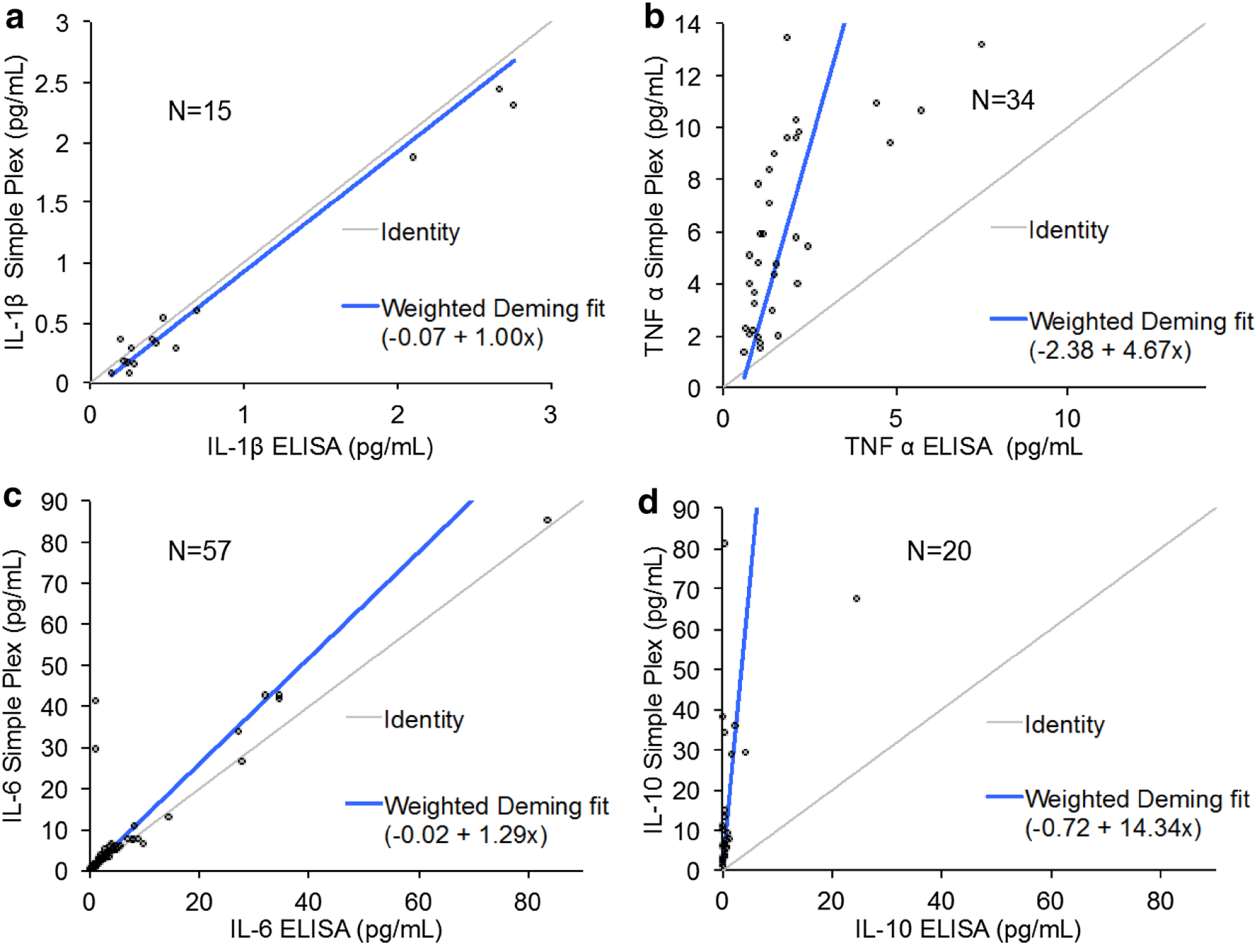
Method comparison between the ELISAs and the Simple Plex assays using Deming regression for measurement of IL-1β (**a**), TNF-α (**b**), IL-6 (**c**), and IL-10 (**d**)

Operational characteristics of Simple Plex, traditional ELISAs, and two other multiplex immunoassay systems (i.e., Bio-Plex^®^ and Multi-Array) are listed in Table 1. Assay imprecisions were evaluated on 6 replicates of duplicate measurements from a pooled serum sample on three different cartridges for IL-1β, TNF-α, IL-6, and IL-10, which were found to be 6.2, 8.0, 6.2 and 9.1 %, respectively. The imprecisions of the Simple Plex assays were comparable to those of the traditional ELISAs, which were evaluated using replicates of the same serum sample pool, to be 9.0, 6.6, 14.5, and 8.3 %, for IL-1β, TNF-α, IL-6, and IL-10, respectively. Table 2 shows the analytical measurement ranges (AMRs) provided by the manufacturer, and the percentages of samples that have results within their respective AMRs, which were 32, 95, 97, and 100 % for IL-1β, TNF-α, IL-6, and IL-10, respectively. The Simple Plex assay did not have the AMR to measure IL-1β levels in 68 % of the 66 samples; that is, 68 % of the samples had IL-1β levels lower than the lower limit of the AMR, which was 0.21 pg/mL (Table 2). We also compared concentration values obtained by the Simple Plex assays and ELISAs in measurement of IL-1β, TNF-α, IL-6, and IL-10 (Fig. 1). Not all 66 samples had measurable results by these assays. For IL-1β and IL-6 assays, the Simple Plex assays used the same antibody pairs as the ELISAs, and demonstrated good correlations by Deming regression, with R^2^ of 0.99 and 0.98, for IL-1β and IL-6, respectively (Fig. 1a, c). For samples with measurable results by both ELISA and Simple Plex, poor correlations between the Simple Plex assays and the ELISAs were observed for TNF-α (n = 34) and IL-10 (n = 20), presumably due to different antibodies used by the different manufacturers (i.e., ProteinSimple and Bio-Techne) in these two assays (Fig. 1b, d).

**Table 1:** Operational characteristics of the Simple Plex device in comparison with peer immunoassay systems

Method	ELISA	Bio Plex	Multi-array	Simple Plex
Manufacturer	Bio-Techne	BioRad	Mesoscale discovery	ProteinSimple
Number of analytes	Single analyte	Up to 100 anaytes	Up to 12 analytes	Up to 8 analytes
Format	96-well plate	96-well plate	96-well plate	Cartridge pre-filled with reagents
Detection	Absorbance	Fluorescence	Electrochemiluminescence	Fluorescence
Capacity (# samples/run)	96	96	96	16
Assay time	4–6 h/run	4-6 h/run	4-6 h/run	1.5 h/run
Level of automation (low, medium, high)	Low	Medium	Medium	High
Volume needed	600 µL^a^	25 µL	25 µL	25 µL

^a^Minimal volume needed to perform 4 ELISAs for measurement of IL-1β, TNF-α, IL-6, and IL-10 in singleton

**Table 2:** The ability of the Simple Plex assays to detect IL-1β, TNF-α, IL-6, and IL-10 in real clinical samples

Cytokine	AMR (pg/mL)^a^	Results within AMR (%)	Results below AMR (%)
IL-1β	0.21–2000	32	68
TNF-α	1.31–5000	95	5
IL-6	0.52–2000	97	3
IL-10	0.21–2000	100	0

^a^AMRs of the Simple Plex assays were provided by the manufacturer

## Discussion

The classical approach for quantitating proteins is generally based on immunoassays, particularly ELISAs. However, ELISAs are typically low throughput, require large sample volumes, and have a relatively high cost due to labor and large amount of reagents consumed (Table 1), especially when they are done manually. To circumvent some of these issues, multiplex immunoassays have been developed, which include planar micro-spot based (e.g., multi-array from meso scale discovery) and bead-based detections (e.g., Bio-Plex^®^ from Bio-Rad) to increase throughput (i.e., multiple analytes vs. single analyte) and conserve sample volume [23–25] (Table 1). One significant difference between the multiplex immunoassays and ELISAs is the fact that, in the planar micro-spot based and bead-based multiplex immunoassays, all antibody pairs required to measure the proteins of interest need to be present in the reagent; whereas in the ELISAs, only one antibody pair is present in the reagent. Ideally, antibodies should only bind to their respective antigens, and vice versa. However, in reality, antibodies bind to not only their respective antigens, but also other molecules present in a sample. Therefore, presence of multiple antibody pairs increases the possibility of antibodies reacting with each others, giving rise to potential off-target effects [12]. Microfluidic-based approaches to multiplexed immunoassay, such as Simple Plex, is an appealing alternative because the design is such that each antibody pair is isolated in its own unit and therefore, completely eliminates cross talk [13, 21]. More importantly, such design would allow each analyte to be optimized (i.e., the amount of antibody, and the dilution factor of the sample, and sample volume) in order to achieve the sensitivity and AMR needed for a specific application.

Currently, the Simple Plex is more automated than either BioPlex^®^ or multi-array assays, whereas the Bio-Plex^®^ or multi-array assays still require several manual steps, such as adding reagents, washing the plates, and reading the plates before results can be obtained. For the Simple Plex device, users only need to pipet samples into the cartridge, insert the cartridge into the analyzer, and hit the “go” button to obtain results. Reagents are incorporated within the cartridges, and no calibrators are needed as cartridges are pre-calibrated by the manufacturer. Because of this higher level of automation (i.e., adding reagents, washing and reading plates), the total analysis time of Simple Plex assays was found to be much shorter than either Bio-Plex^®^ or Multi-Array assays (1.5 h versus 4–6 h) (Table 1). However, the Simple Plex analyzes 16 samples in a cartridge, less than 96 samples that the Bio-Plex^®^ and Multi-Array assays would be able to analyze in a 96 well plate. It uses 25 μL sample, the same as Bio-Plex^®^ and Multi-Array assays. Therefore, in order to offer more advantageous features, the Simple Plex device should consider improving its throughput (16 samples per cartridge) and decreasing required sample volume (25 μL).

A frequent constraint inherent to miniaturization and microfluidics is the low signal-to-noise ratio and the low detectability of the analytical signals [15]. Therefore, many publications involving microfluidic methods alone do not describe their applications to the analysis of real serum samples. The use of nanomaterials in microfluidics is a recent trend to improve sensitivity and selectivity, which is crucial in measurement of real clinical samples. Nanomaterials have been used in different steps of the analytical process of a microfluidic device: preconcentration, separation, reaction, and detection [15]. Nanomaterials offer properties such as large surface area-to-volume ratio and relative easy functionalization for preconcentration and separation steps (e.g., by coupling to antibodies or other biomolecules). Also, nanomaterials have been used as buffer additives and as stationary phases in microchip electrochromatography [26]. Furthermore, nanomaterials offer catalytic properties and capability to act as electron-transfer mediators to improve analytical reaction in microfluidic methods [27]. Last but not least, nanomaterials are used in the detection step to improve the sensitivity of microfluidic methods [15]. Simple Plex assays use GNRs, which are hollow, cylindrical reaction chambers composed of fused silica, in improving selectivity and sensitivity in separation and detection steps. These porous silica nanoreactors are functionalized to offer a highly uniformed, solid phase immobilization of detection antibodies on the internal surface of the nanoreactor. The optical properties of the glass nanoreactors are ideally suited for subsequent fluorescent detection (i.e., low intrinsic fluorescence) [21].

Our results in applying the Simple Plex methodology to real clinical samples, randomly selected from our Endocrine Laboratory, showed that the IL-1β assay did not have enough sensitivity (i.e., the low limit of the AMR) for 68 % of the 66 samples analyzed (Table 2). Future assay generations may address this lack of sensitivity by designing a micro-fluidic cartridge where splitting the specimen into different volumes so that the volume for IL-1β assay will be set optimally for the purpose of improving sensitivity. Alternatively, chemiluminescent detection, which usually is more sensitive than fluorescence detection, may be another way to increase the sensitivity of this device [28].

The Simple Plex device embodies a current trend of combining microfluidics with nanotechnology for simultaneous measurement of protein biomarkers. Our study illustrated the significant advantages of the Simple Plex device over traditional multiplex immunoassay systems in its miniaturization and automation analysis. Despite the limitation in sensitivity for certain analyte (i.e., IL-1β), microfluidic devices such as Simple Plex represent a promising technological platform to be used in translational research for simultaneous measurement of multiple protein biomarkers in real samples.

## Abbreviations

IL-1β: Interleukin-1 beta
IL-6: Interleukin-6
IL-10: Interleukin-10
TNF-α: Tumor necrosis factor-α
CV: Coefficients of variation
AMR: Analytical measurement range
QC: Quality control
